# Lysosomal gene ATP6AP1 promotes doxorubicin resistance via up-regulating autophagic flux in breast cancer

**DOI:** 10.1186/s12935-024-03579-9

**Published:** 2024-12-03

**Authors:** Yinjiao Fei, Xueqin Yan, Mingxing Liang, Shu Zhou, Di Xu, Lei Li, Weilin Xu, Yuxin Song, Zhen Zhu, Jian Zhang

**Affiliations:** 1 Department of Radiation Therapy, the First Affiliated Hospital With Nanjing Medical University, 300 Guangzhou Road, Nanjing, 210029 People’s Republic of China; 2 Department of General Surgery, the First Affiliated Hospital With Nanjing Medical University, 300 Guangzhou Road, Nanjing, 210029 People’s Republic of China; 3 Department of Thyroid Surgery, the Second Affiliated Hospital of Zhejiang University School of Medicine, 88Jiefang Road, Hangzhou, 310009 People’s Republic of China

**Keywords:** Breast cancer, Doxorubicin, ATP6AP1, Autophagy, Chemoresistance

## Abstract

**Background:**

Breast cancer remains the most prevalent malignancy in women. Chemotherapy is the primary systemic treatment modality, and the effectiveness of treatment is often hampered by chemoresistance. Autophagy has been implicated in promoting chemoresistance, as elevated autophagic flux supports tumor cell survival under therapeutic stress. Since lysosomes are essential for the completion of autophagy, their role in autophagy-related chemoresistance has been insufficiently studied. This study aims to elucidate the role of the lysosomal gene ATP6AP1 in promoting chemoresistance in breast cancer by upregulating autophagic flux.

**Methods:**

Doxorubicin-induced cell death was assessed by cytotoxicity, flow cytometry, lactate dehydrogenase (LDH) release assays in various breast cancer cell lines. Autophagic flux was assessed with western blot and the mRFP-GFP-LC3 fluorescence imaging. Breast cancer cells were infected with shRNA lentivirus targeting ATP6AP1, allowing investigation its tole in doxorubicin-induced cell death. ATP6AP1 expression and its association with prognosis were evaluated using public databases and immunohistochemistry.

**Results:**

Doxorubicin-induced cell death in breast cancer cells is negatively correlated with increased autophagic flux and lysosomal acidification. The lysosomal gene ATP6AP1, which plays a role in autophagic processes, is upregulated in breast cancer tissues. Knocking down ATP6AP1 reduces autophagy-mediated doxorubicin resistance by inhibiting autophagic flux and lysosomal acidification in breast cancer cells. Data analysis from public databases and our cohort indicate that elevated ATP6AP1 expression correlates with poor response to doxorubicin-based neoadjuvant chemotherapy (NAC) and worse prognosis.

**Conclusions:**

Doxorubicin-induced cytotoxicity is associated with autophagy flux in breast cancer. The lysosomal gene ATP6AP1 facilitates autolysosome acidification and contributes to doxorubicin resistance in breast cancer.

**Supplementary Information:**

The online version contains supplementary material available at 10.1186/s12935-024-03579-9.

## Introduction

Breast cancer is the prevalent malignancy among women worldwide and ranks as the second leading cause of cancer-related mortality [1]. Among the primary therapeutic modalities, systemic chemotherapy remains integral to the treatment landscape, aiming to improve survival outcomes and manage disease progression [2]. However, the doxorubicin (DOX) resistance poses a significant hurdle, undermining the efficacy of these treatments and underscoring the pressing demand for groundbreaking approaches to boost DOX efficacy in cancer cells.

Autophagy, an indispensable cellular process, orchestrates the catabolism and recycling of cellular constituents in both normal and stress-provoked contexts, such as those elicited by chemotherapeutic agents [3]. Central to autophagic functionality is the lysosomal apparatus, which plays an essential role in the degradation of malfunctional cellular components within its acidic milieu [4–7]. Significantly, DOX has been delineated as a robust inducer of autophagy, as evidenced by the increased levels of LC3-II, p62, and Beclin1 proteins [8, 9]. However, the involvement of autophagy in the context of DOX-induced cell death remains contentious. The upregulation of autophagy in response to DOX treatment facilitates cellular capacity to remove damaged components, thereby promoting a resistance mechanism that reduces DOX’s therapeutic efficacy [10–12]. Recent evidences elucidate that impediments in autophagic flux and lysosomal acidification culminate in the accumulation of autophagosomes, subsequently precipitating reactive oxygen species generation and DOX-mediated cytotoxicity [13, 14], suggesting that the lysosomal acidification process may be involve in the DOX-induced cell death and resistance.

The vacuolar-type H+-ATPase (V-ATPase) transports protons into the lysosome, establishing an acidic pH gradient that is necessary for activating of lysosomal hydrolases [15, 16]. Perturbations in the expression or functionality of V-ATPase lead to reduced lysosomal acidification, thereby adversely affecting both the fusion of autophagosomes with lysosomes and the lysosomal degradative capacity [17–19]. The targeted knockdown of the V-ATPase subunit ATP6L via siRNA increases the sensitivity of DOX-resistant breast cancer cells to DOX cytotoxicity [20], suggesting that lysosome-associated proteins, particularly those involved in lysosomal acidification, may play a critical role in the modulation of DOX resistance. ATP6AP1 (ATPase H+ Transporting Accessory Protein 1), also known as Ac45, functions as a crucial assembly factor for V-ATPase, facilitating the integration of multiple subunits and phospholipids during the assembly process [21]. Mutations or insufficiencies in ATP6AP1 are associated with an array of V-ATPase-related disorders, encompassing immunodeficiency with hepatopathy, neurological features, distal renal tubular acidosis, and cancer [22–24], underscoring its indispensable role in autophagy through the maintenance of an acidic milieu within lysosomes [25]. However, the precise contribution of ATP6AP1 to lysosomal acidification and its influence on DOX-induced cell death is yet to be clarified.

In this study, we have demonstrated that the DOX-induced autophagic flux contributes to DOX resistance in breast cancer. Through comprehensive analysis of autophagy-lysosomal gene expression, we identified that the lysosome-associated gene ATP6AP1 is consistently upregulated in breast cancer tissues and associated with worse clinical outcomes. Importantly, knockdown of ATP6AP1 blocks DOX-induced autophagic flux and partially reverses DOX resistance in breast cancer cells. These findings suggest that the lysosomal function is essential for DOX-induced autophagy flux and subsequent chemoresistance, modulating lysosome-associated proteins like ATP6AP1, might improve the cytotoxicity of DOX in breast cancer cells.

## Materials and methods

### Data acquisition and collection

The transcriptome expression data, along with detailed clinicopathologic information, were sourced from the Gene Expression Omnibus (GEO) database (http://www.ncbi.nlm.nih.gov/geo) and The Cancer Genome Atlas (TCGA). In cases where multiple probes were available, the mean expression was utilized. The mRNA expression and clinical data of the Molecular Taxonomy of Breast Cancer International Consortium (METABRIC) dataset were retrieved from cBioPortal (https://www.cbioportal.org/). Genes difference across the normal and tumor tissues was analyzed using the Limma, differentially expressed genes exhibiting significant changes (Log2 Fold Change > 1, p-value < 0.05, and FDR < 0.05) in TCGA-BRAC cohort.

### Cell lines and culture conditions

MCF-10A, MCF-7, T-47D, ZR-75-1, BT-474, SK-BR-3, MDA-MB-453, MDA-MB-231, BT-549, Hs 578T, MDA-MB-468 and HCC1806 cells were purchased from Cell Bank of Chinese Academy of Medical Science (Shanghai, China). SUM1315MO2 was from Dr. Stephen Ethier (University of Michigan, USA). The MCF-10A cell line was cultured in complete medium (CM-0525, Procell, China). Other cells were cultured in RPMI 1640 medium (KGM31800-500, KeyGEN, China) supplemented with 10% of fetal bovine serum (086-150, WISENT, China) at 37 °C in a humidified atmosphere of 95% air and 5% CO_2_. Before conducting cell experiments, we replaced the culture medium with an antibiotic-free medium. Knockdown (shATP6AP1) and control vector viral particles were produced using 293 T cells followed by the transduction to MDA-MB-453 and T-47D cells. Cells were selected using puromycin (2 μg/mL, P8833, Sigma-Aldrich, USA) for seven days. The shRNA sequences were as followed: shATP6AP1-1, ACAGTGACATTCAAGTTCATT; shATP6AP1-1, GCATTGAGGATTTCACAGCAT.

### Western blot assay

Total protein was extracted using cell lysis buffer (P0013B, Beyotime) supplemented with PhosSTOP (4906845001, Roche, Indianapolis, IN) and cOmplete (11836170001, Roche). Total protein lysates (20 μg) were analyzed with SDS-PAGE and transferred to polyvinylidene difluoride membranes (Millipore, Billerica, MA, USA). The membranes were blocked with 5% skimmed milk for 1h, and then incubated overnight at 4°C with primary antibodies against PARP-1(1:1k, sc-7150, Santa Cruz Biotechnology,), p62(1:1k, A7758, Abclonal), ATP6AP1(1:1k, sc-81886, Santa Cruz Biotechnology,), LC3 (1:1k, 3868, CST), GAPDH (1:3000, 92310, CST,). After washing with TBST, the membranes were incubated with Streptavidin-HRP for 1 h and detected with the enhanced chemiluminescence reagents.

### Analysis of autophagic flux

To monitor the autophagic flux, breast cancer cells were transfected with pLV3-CMV-mCherry-EGFP-LC3B reporters (P48062, MiaoLingBio, China) for 48 h and then treated with DOX for additional 24 h. The LC3-GFP or LC3-mCherry images were acquired using fluorescence microscopy (Carl Zeiss AG, Axio Vert. A1, Germany).

### Cytotoxicity and lactate dehydrogenase (LDH) activity assay

For cytotoxicity assay, 5 × 10^3^ cells of different groups were seeded in 96-well plates. After adhesion, the cells were treated with or without DOX (2μM, HY-15142, MCE, China), Bafilomycin A1 (Baf-A1,100nM, HY-100558, MCE) or Chloroquine (CQ,10μM, HY-17589A, MCE) for 48 h. Cell viability was determined by CCK-8 (C0038, Beyotime, China) with a microplate reader (CMAX PLUS, Molecular Devices, China). The LDH release in supernatant was detected with LDH Cytotoxicity Assay Kit (C0016, Beyotime).

### Flow cytometry

1 × 10^6^ breast cancer cells were seeded in 6-well plates and treated with or without DOX (2 μM), Baf-A1 (100nM) or CQ (10 μM) for 48 h. Cell death was measured using Annexin V-FITC/PI Apoptosis Detection Kit (A211, Vazyme, China). According to the instructions, cells were stained with Annexin V-FITC and PI Staining Solution for 10 min. Flow cytometry excitation was performed by CytoFLEX (Beckman, USA).

### Immunohistochemistry

Immunohistochemistry (IHC) was performed using IHC staining kit (KIT-9710, Maxim, China). The antibodies against ATP6AP1 were obtained from Proteintech Group (1:100, 15305-1-AP). DAB Kit (DAB-2031, MXB) was applied to show the staining results and mixed according to manufacturer’s instructions to expose staining difference. IHC scores were determined using semi-quantitative optical analysis by the light microscopy (AX10, Zeiss, Germany). The percentage of positive cells (staining percentage score, based on the percentage score of stained positive cells 1–4:1 = 0–25%, 2 = 25–50%, 3 = 50–75%, 4 = 75–100%) and staining intensity (intensity score, score 1–3:1 = weak, 2 = medium, 3 = strong) were calculated. The final IHC score was obtained by multiplying the percent staining score with the intensity score. The samples were scored by two independent pathologists who were blinded to patient characteristics.

### Clinical samples and clinical characteristics

This project was approved by the Ethics Committee of the First Affiliated Hospital of Nanjing Medical University (2022-SR-520). Nineteen pairs of fresh breast cancer and adjacent tissues (IHC cohort I, Table S1) and fifty paraffin-embedded breast cancer sample slices (IHC cohort II, Table S2) were acquired from the First Affiliated Hospital of Nanjing Medical University. Informed consent was obtained from all patients before tissue collection.

### Statistical analyses

All results were repeated at least three independent experiments and at least triplicates were plotted with mean with standard deviations (SD). Kaplan–Meier curves and log-rank tests for survival analyses, Pearson correlation, two-tailed Student’s *t*-test and Fisher’s exact test between variables were performed by GraphPad Prism 8.0 software (*, p < 0.05; **, p < 0.01; ***, p < 0.001).

## Results

### Autophagy attenuates DOX-induced cell death in breast cancer cells

**Fig. 1:**
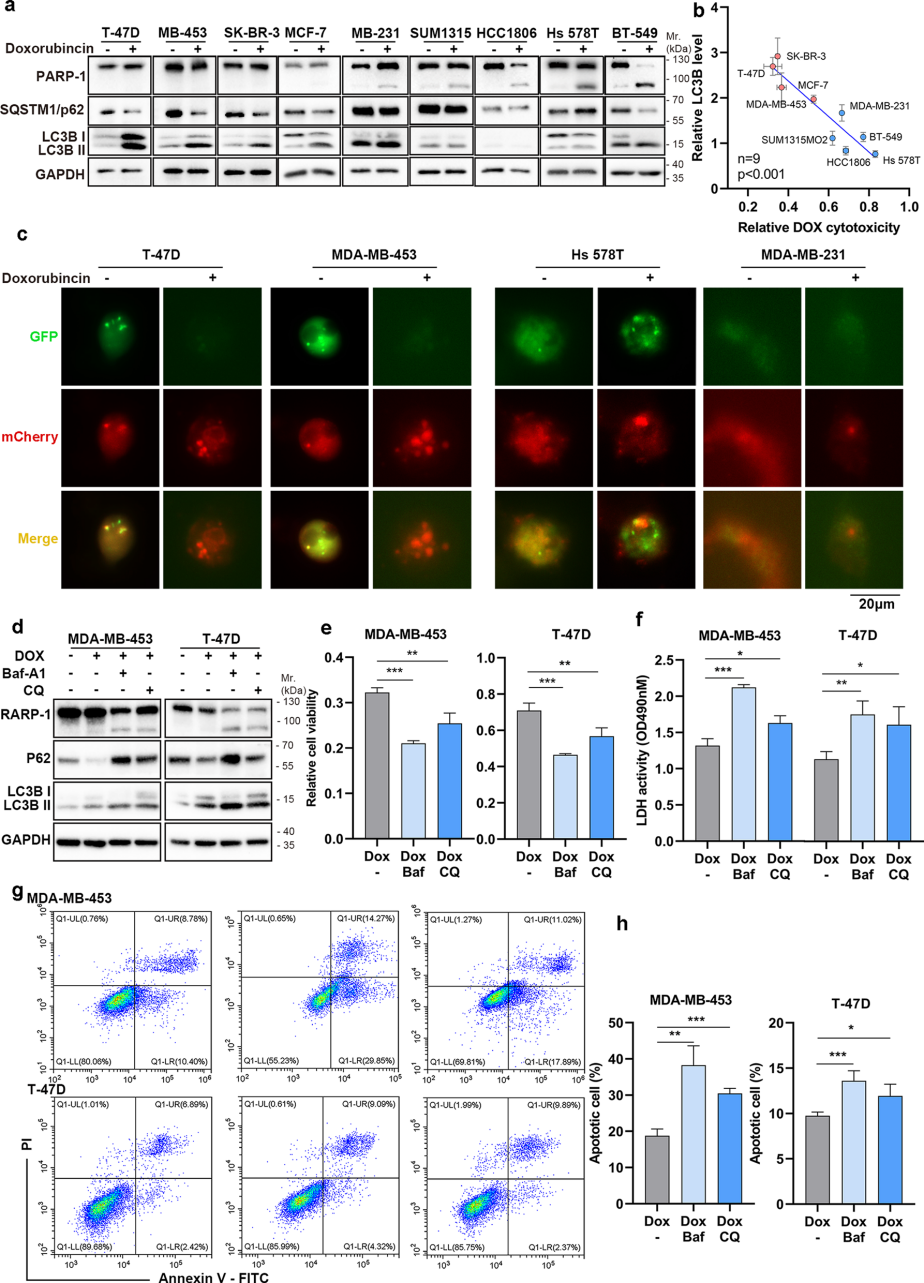
Autophagy is associated with DOX-induced cytotoxicity in breast cancer cells. **a** The protein levels of PARP-1, LC3 and p62 in nine breast cancer cell lines treatment with DOX (2μM). **b** The association of LC3B II protein level and relative DOX cytotoxicity. The LC3B protein level were assessed by western blot and normalized to cells without DOX treatment. The relative DOX cytotoxicity was generated from CCK-8. **c** T-47D, MDA-MB-453, Hs 578T and MDA-MB-231 were transfected with mCherry-GFP-LC3 reporter with or without DOX treatment. The LC3-GFP or LC3-mCherry were picture by a fluorescence microscopy. Scale bars, 20 µm. **d** The protein level change of T47D and MDA-MB-453 cells treated with DOX, Baf-A1(100 nM) and CQ (10 μM). **e**–**h** DOX-induced cytotoxicity were assessed by relative cell viability (**e**), extracellular LDH activity (**f**) and Annexin V/PI staining (**g**, **h**). Cells were with or without treated with Baf-A1 and CQ. The triplicate experiments were plotted with mean ± SD, *, p < 0.05, **, p < 0.01, ***, p < 0.001

To elucidate the association of autophagy and DOX-induced cell death, we investigated the sensitivity to DOX and DOX-induce autophagy in nine breast cancer cells. Specifically, T-47D, SK-BR-3, MCF-7, and MDA-MB-453 exhibited relative insensitivity to DOX, while MDA-MB-231, Hs-578T, BT-549, HCC1806 and SUM1315MO2 displayed relative sensitivity to DOX-induced cell death as evidence by western blot and cytotoxicity assays (Fig. 1a and b). We also found the negative association of DOX-induced autophagy (as evidenced by relative protein level of LC3B II) and DOX-induce cytotoxicity (evidenced by cytotoxicity assays) upon DOX treatment in breast cancer cell lines (Fig. 1a and b). To depict the autophagic flux difference in DOX sensitive and resistance, T-47D, MDA-MB-453, Hs-578T and MDA-MB-231 with mCherry-GFP-LC3 plasmid transfection. In cells treated with DOX, LC3-mCherry puncta were increased, however the LC3-GFP puncta were loss in T-47D and MDA-MB-453 cells, which indicates the acidification of autophagosome in these cells but not in Hs-578T and MDA-MB231 cells (Fig. 1c). To confirm the association of happen of autophagy flux and DOX resistance, T-47D and MDA-MB-453 cells were treated with the lysosome acidification inhibitor Bafilomycin-A1 (Baf-A1) or autophagosome-lysosome fusion inhibitor chloroquine (CQ). The results showed that when treated with DOX alone, there was no significant activation of PARP-1 in these cells, however, administration Baf-A1 or CQ led to increased cell death (Fig. 1d–h), indicating the autophagy flux inhibition promote sensitive to DOX.

Taken together, autophagy is associated with DOX resistance in breast cancer. Specifically, autophagosome-lysosome process mediates DOX resistance.

### Lysosome related gene ATP6AP1 is upregulated in breast cancer tissues

**Fig. 2:**
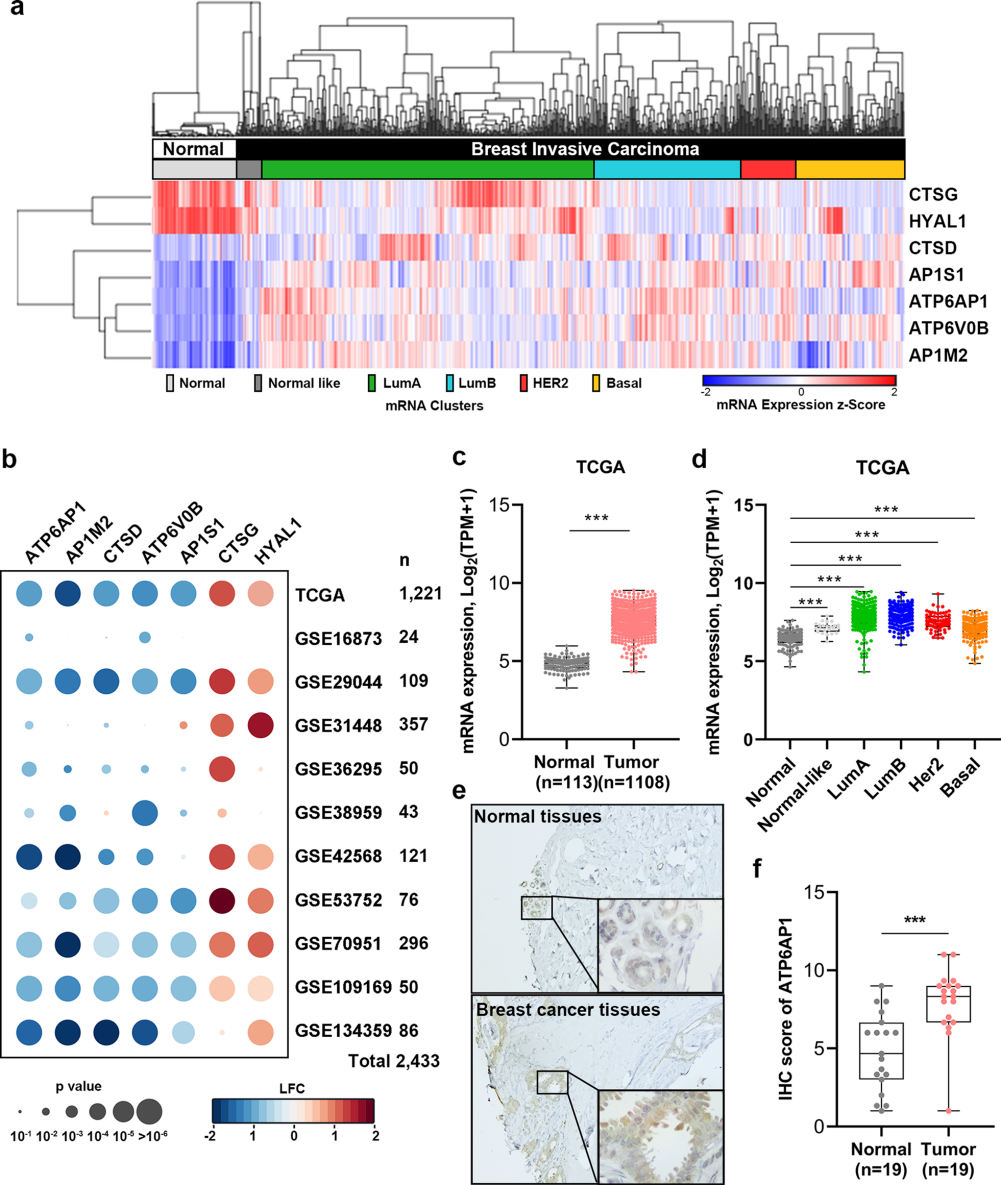
ATP6AP1 is upregulated in breast cancer tissues. **a** Hierarchical clustering illustrates the 7 differentially-expressed lysosome-related genes in TCGA-BRCA between UBC tissues and the adjacent normal tissues. **b** Heatmap showed the association of differentially-expressed lysosome-related genes in tumor with normal tissues across multiple EC cohorts. Colored-blocks in the heatmap represented the lysosome-related genes with upregulation (in red) or downregulation (in blue), respectively. LFC, Log2 fold change. **c** and **d** The mRNA expression profile of ATP6AP1 in breast cancer and normal tissues (**c**) and different molecular subtypes (**d**) in the TCGA database. **e** and **f** IHC staining (**e**) and statistics (**f)** of ATP6AP1 in breast cancer and the adjacent normal tissues. Representative images illustrating different staining intensities are presented in panel (**e**). Scale bars, 400 µm (upper panel) and 20 µm (lower panel). *, p < 0.05, **, p < 0.01, ***, p < 0.001

To explore the potential prognostic value of lysosome related genes in breast cancer patients, we first surveyed the expression of these genes between tumor and normal tissues in TCGA dataset. Based on the KEGG and GO pathway, there are 126 gene involved in lysosome pathway. Among all lysosome related genes, 7 genes were differently expressed between breast cancer tissues and noncancerous tissues (p < 0.0001, Fig. 2a). Among 7 genes, ATP6AP1 (ATPase H+ Transporting Accessory Protein 1) showed a consistent upregulation in tumor tissues across multiple cohorts and TCGA cohort (Fig. 2b and c). Interestingly, we also found that upregulation of ATP6AP1 was found among molecular subtypes of breast cancer specifically in luminal subtypes (Fig. 2d), which were known with poor chemotherapy response in breast cancer [26]. To further validate its upregulation in breast cancer samples, immunohistochemistry (IHC) staining from cohort I showed that ATP6AP1 notably expressed in the cytoplasm of cancer cells, but weakly positively stained in the normal tissues (Fig. 2e and f). In summary, we concluded that ATP6AP1 was frequently increased in breast cancer.

### ATP6AP1 knockdown increases DOX-induced cell death by inhibiting autophagy flux

**Fig. 3:**
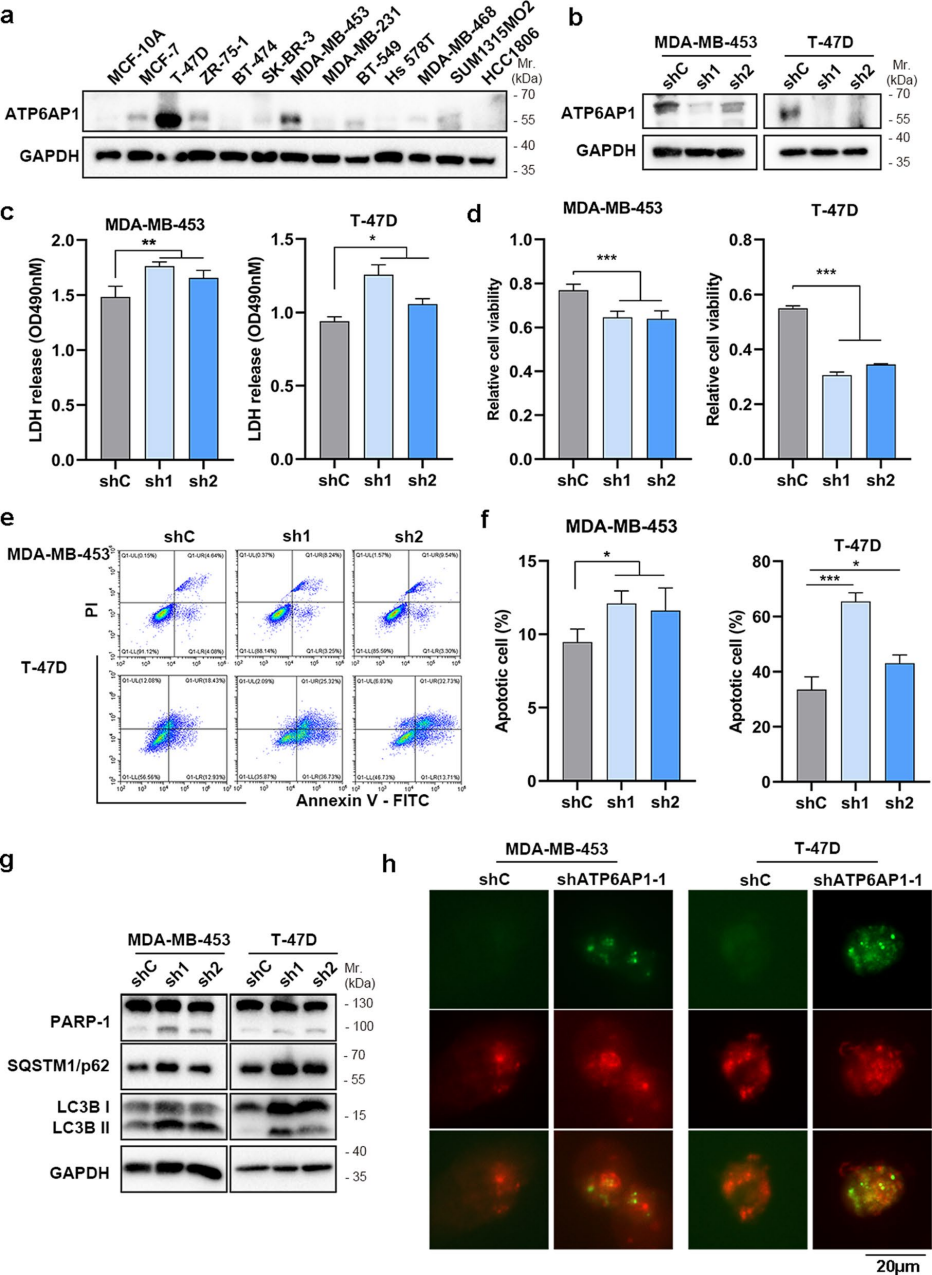
ATP6AP1 promotes DOX resistance in breast cancer cells. **a** The protein levels of ATP6AP1 in 12 breast cancer cells and MCF-10A were determined by western blot assay. **b** Knockdown of ATP6AP1 in MDA-MB-453 and T-47D cells were confirmed by western blotting. **c-f** Effects of ATP6AP1 knockdown on extracellular LDH activity (**c**), relative cell viability (**d**) and Annexin V/PI staining (**e**–**f**) of MDA-MB-453 and T-47D cells treatment with DOX (2 μM). **g** The apoptosis and autophagy related proteins were analyzed by western blotting. **h** The autophagy and lysosome fusion reporter were pictured LC3-GFP or LC3-mCherry, Scale bars, 20 µm. Data are presented as means ± SD. *, p < 0.05, **, p < 0.01, ***, p < 0.001

Given autophagy flux especially the autophagosome-lysosome fusion process association with DOX resistance and higher expression of lysosome related gene ATP6AP1 in breast cancer tissues, we investigated association of ATP6AP1 with DOX resistance. As shown in Fig. 3a, ATP6AP1 was primarily found at high levels of expression in breast cancer cell lines (MCF-7, T-47D, ZR-75-1 and MDA-MB-453). In contrast, its expression was less pronounced in normal breast cells (MCF-10A). To investigate the potential role of ATP6AP1 in the chemosensitivity of breast cancer cells, we stably knocked down ATP6AP1 in T-47D and MDA-MB-453 cells (Fig. 3b). Notably, ATP6AP1 knockdown resulted in higher LDH release (Fig. 3c), lower cell viability (Fig. 3d) and higher cell death (Fig. 3e and f) upon on DOX treatment. Western blot analysis showed a significantly increase of cleaved PARP and autophagy maker LC3B and SQTM1/p62 (Fig. 3g), which is resemble to the result of administration of CQ and Baf-A1 (Fig. 1d). Autophagy flux analysis showed that the treatment of DOX results in a loss of LC3-GFP puncta signal, but the knockdown of ATP6AP1 conserved the LC3-GFP puncta signal in MDA-MB-453 and T-47D cells, which indicated that the knockdown of ATP6AP1 prevent the acidification of autophagosome result in an autophagy arrest and DOX cytotoxicity (Fig. 3h).

In summary, these findings suggest that the downregulation of the ATP6AP1 leads to a weakened cellular autophagy flux, enhancing cell cytotoxicity induced by DOX.

### ATP6AP1 is associated with poor response to DOX based chemotherapy and prognosis

**Fig. 4:**
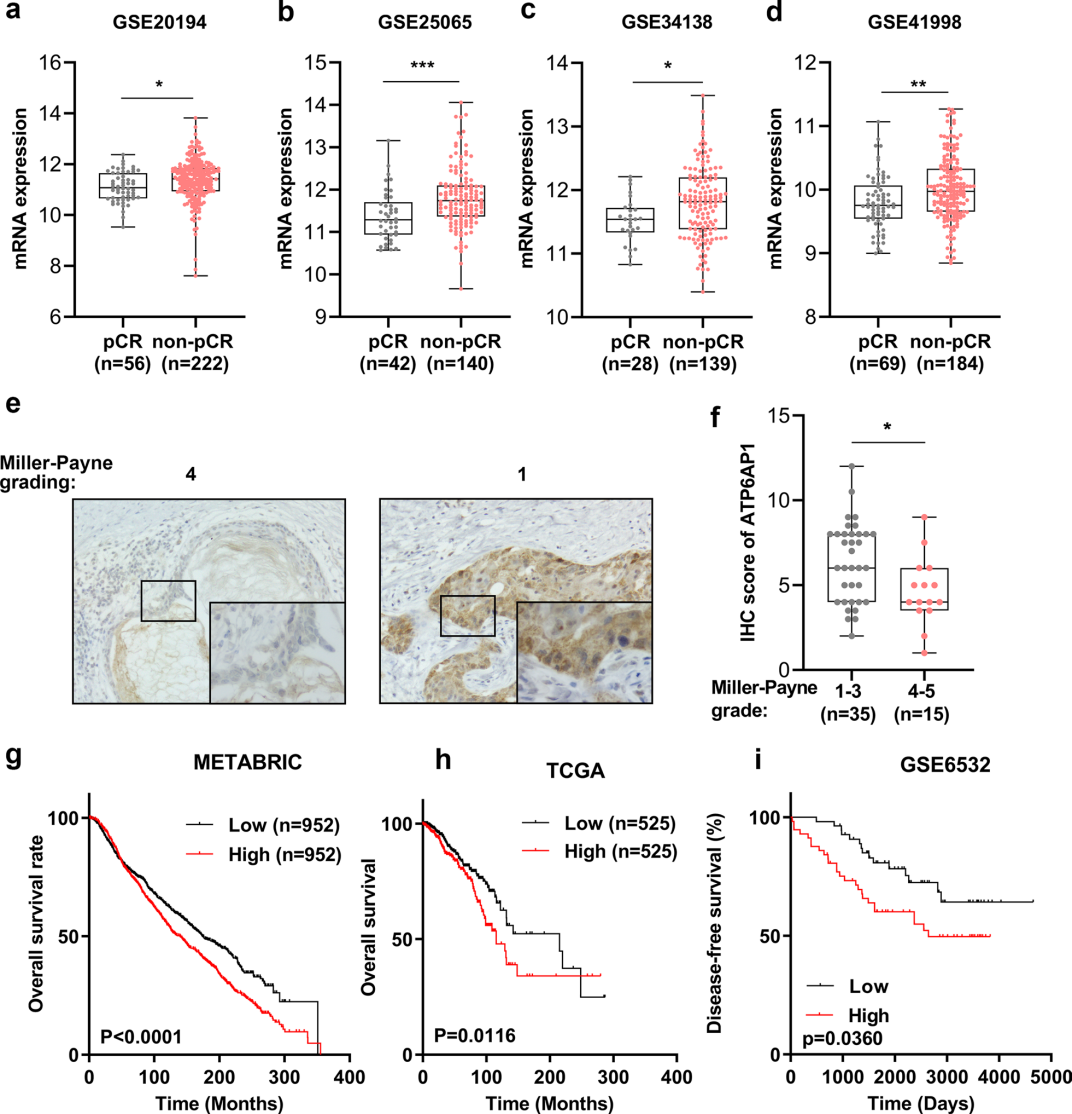
ATP6AP1 is associated with poor clinical response to DOX based chemotherapy and prognosis. **a**–**d** The mRNA expression of ATP6AP1 in GEO cohorts with DOX based chemotherapy. **e**, **f** The IHC staining (**e**) and statistics (**f**) of ATP6AP1 in patients with neoadjuvant chemotherapy. The representative images for different staining intensities are shown (**e**). Scale bars, 200 µm (out) and 20 µm (inset). **g**–**i** Kaplan–Meier plot of overall survival of ATP6AP1 in METABRIC (**g**) and TCGA-BRCA (**h**), and Kaplan- Meier plot of disease-free survival of ATP6AP1 in GSE6532 (**i**). *, p < 0.05, **, p < 0.01, ***, p < 0.001

To investigate the association of ATP6AP1 with DOX resistance clinically, we analysis the GEO cohorts, including GSE20194, GSE25065, GSE34138 and GSE41998 cohorts with DOX based neoadjuvant chemotherapy. A significantly lower expression of ATP6AP1 patients with pathological complete response (pCR) in these cohorts (GSE20194, p < 0.05, GSE25065, p < 0.001, GSE34138, p < 0.05, GSE41998, p < 0.01, Fig. 4a–d). These findings were consistent with our immunohistochemistry (IHC) results from cohort II, indicating a substantial lower ATP6AP1 expression in patients showing pathological response to DOX based neoadjuvant therapy (MP score < 4, Fig. 4e, f and Table S2). Additionally, patients with high ATP6AP1 expression had poorer overall survival in METABRIC and TCGA cohorts (Fig. 4g, h). Moreover, in the GSE6532 database, patients with high ATP6AP6 expression also exhibited a worse disease-free survival period (Fig. 4i).

These data indicated that the lower expression of ATP6AP1 is associated with pathological complete response in DOX based neoadjuvant chemotherapy in breast cancer. The higher expression of ATP6AP1 might be a druggable target for DOX resistance in breast cancer.

## Discussion

DOX currently holds a pivotal role as one of the foremost chemotherapy drugs and is acknowledged as a primary choice for systemic breast cancer treatment [27]. Nevertheless, resistance to Dox, stemming from intrinsic or acquired drug resistance mechanisms, is extensively documented in breast cancer [28, 29]. Amidst various contributing factors, autophagy, which acts as a protective mechanism, assumes a crucial role in the development of chemotherapy resistance [30]. Numerous studies have highlighted that DOX treatment can induce chemotherapy resistance by activating autophagy across various cancer types [31–33]. Consequently, the inhibition of autophagy emerges as an effective strategy to overcome or reverse DOX resistance, thereby augmenting the overall efficacy of chemotherapy.

We first evaluated the autophagic activity in various breast cancer cells following DOX treatment. We observed an upregulation in the expression of the autophagy-related protein LC3 II and the degradation of P62 in breast cancer cells insensitive to DOX. Additionally, the mRFP-GFP-LC3 construct consistently indicated the autophagic flux changing in the DOX-resistance breast cancer cells. Significantly, the co-administration of common autophagy inhibitors, such as CQ and Baf-A1, with DOX, induced apoptosis and reversed doxorubicin resistance in breast cancer cells. These findings underscore the pivotal role of autophagy in influencing the sensitivity of breast cancer cells to chemotherapy.

ATP6AP1, also known as Ac45, is a protein that plays a crucial role in regulating intraorganellar pH. It is associated with the ATPase H+ (V-ATPase) complex, which is responsible for maintaining the acidic environment within lysosomes [34, 35]. Proper lysosomal acidification is essential for the activation of lysosomal enzymes involved in various cellular processes, including autophagy [36]. Recent studies have suggested that ATP6AP1 could serve as a therapeutic target for osteolytic diseases and colorectal cancer [37, 38]. Additionally, salivary autoantibodies against ATP6AP1 have been explored as potential tools for efficiently screening early cases of breast cancer [39]. However, it is currently unclear whether ATP6AP1 regulates autophagy in breast cancer cells undergoing chemotherapy and the specific impact of ATP6AP1 on chemotherapy resistance in breast cancer. Therefore, to improve the survival rate of breast cancer patients and overcome chemotherapy resistance, our focus is on the interplay between ATP6AP1 and autophagy.

In this study, we demonstrated that ATP6AP1-mediated autophagy played a critical role in breast cancer chemoresistance. Through a comprehensive analysis of autophagy-lysosomal gene expression profiles, we identified ATP6AP1 as a key regulator of autophagy in breast cancer cells, contributing significantly to resistance against DOX. Elevated ATP6AP1 expression was consistently observed in breast cancer subtypes associated with poorer clinical outcomes, particularly in patients showing reduced responsiveness to neoadjuvant chemotherapy. Our data further revealed that knocking down ATP6AP1 in breast cancer cells significantly increased sensitivity to DOX by inhibiting autophagy. This aligned with previous findings highlighting autophagy’s protective role in enabling cancer cell survival under chemotherapy-induced stress. ATP6AP1 depletion disrupted autolysosome formation or function, leading to reduced autophagic flux and rendering cancer cells more susceptible to DOX-induced cell death. These findings underscore the potential therapeutic value of targeting ATP6AP1 or modulating autophagic flux in overcoming chemoresistance.

However, our study has certain limitations. Primarily, the in-depth investigation of mechanisms is relatively insufficient. Although we have preliminarily clarified the role of ATP6AP1-mediated autophagy in breast cancer drug resistance through cell experiments and similar methods, we have not conducted a thorough and comprehensive analysis of its underlying mechanisms. Future research could employ more sophisticated techniques, such as gene editing and protein–protein interaction analyses, to reveal in greater detail and comprehensiveness the exact mechanisms of ATP6AP1 in the development of drug resistance in breast cancer. Additionally, animal experiments are commonly employed to provide a deeper understanding of biological processes, further validating and supporting the results obtained from in vitro experiments. The lack of animal experiments in this study may restrict our comprehension of complex biological reactions and pharmacological effects occurring within living organisms. Future research endeavors could consider incorporating animal models to comprehensively assess the phenomena studied in the context of the overall physiological environment, better simulating real therapeutic scenarios.

## Conclusion

**Fig. 5:**
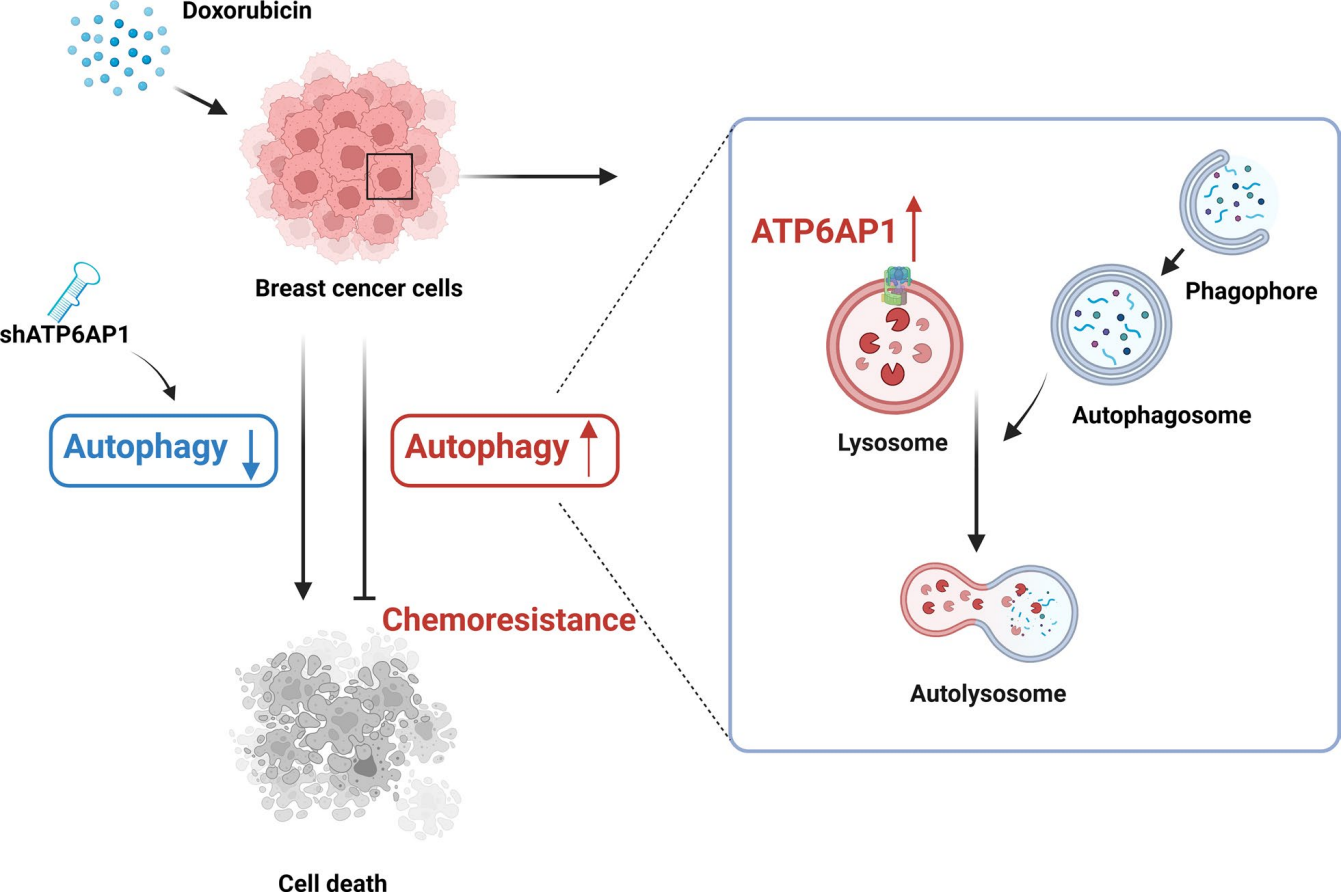
Proposed mechanism of ATP6AP1-mediated chemoresistance in breast cancer. DOX treatment induces autophagy in breast cancer cells, contributing to chemoresistance. ATP6AP1, a lysosome-related gene, is upregulated in breast cancer cells with chemoresistance. Inhibiting ATP6AP1 by blocking autophagy enhances drug sensitivity in breast cancer cells, leading to improved therapeutic outcomes and overall survival of breast cancer patients

In this study, we observed an association between DOX-induced autophagic flux and chemoresistance in breast cancer. Inhibiting ATP6AP1 by blocking autophagy can increase drug sensitivity in breast cancer cells, thereby improving the therapeutic outcomes and overall survival of breast cancer patients (Fig. 5). These results support an investigation of ATP6AP1 as a potential target for breast cancer therapy.

## Supplementary Information

Supplementary Material 1.

## Data Availability

The data underlying this article will be shared on reasonable request to the corresponding author.
