# Supplementary material for: Lysosomal gene ATP6AP1 promotes doxorubicin resistance via up-regulating autophagic flux in breast cancer
Source: Cancer Cell Int. 2024 Dec 3;24:394. doi: 10.1186/s12935-024-03579-9 (PMC11616228; doi:10.1186/s12935-024-03579-9)
Supplement: Supplementary file 1 — Supplementary Material 1. [file 12935_2024_3579_MOESM1_ESM.docx]

**Supplement table**

**Table S1. The association between ATP6AP1 protein levels and clinicopathological features of IHC cohort I (n=19)**

| **Variables** | **n** | **Expression of ATP6AP1** | | **p** |
| --- | --- | --- | --- | --- |
|  |  | **High**  **(n, %)** | **Low**  **(n, %)** |  |
| **Age (years)** | 19 | 49.23±9.976 | 52.50±8.191 | 0.4945 |
| **T stage** |  |  | | 0.0722 |
| 1 | 6 | 4（66.7） | 2（33.3） |  |
| 2 | 11 | 9（81.8） | 2（18.2） |  |
| 3-4 | 2 | 0（0.0） | 2（100.0） |  |
| **N stage** |  |  |  | 0.9990 |
| 0 | 10 | 7（70.0） | 3（30.0） |  |
| 1 | 3 | 2（66.7） | 1（33.3） |  |
| 2 | 3 | 2（66.7） | 1（33.3） |  |
| 3 | 3 | 2（66.7） | 1（33.3） |  |
| **Pathological stage** |  |  |  | >0.9999 |
| I- II | 13 | 9（69.2） | 4（30.8） |  |
| III-IV | 6 | 4（66.7） | 2（33.3） |  |
| **ki67 status** |  |  |  | >0.9999 |
| <30% | 8 | 6（75.0） | 2（25.0） |  |
| ≥30% | 11 | 7（63.6） | 4（36.4） |  |

T, primary tumor; N, regional lymph nodes.

**Table S2. The association between ATP6AP1 protein levels and clinicopathological features of IHC cohort II (n=50)**

| **Variables** | **n** | **Expression of ATP6AP1** | | **p** |
| --- | --- | --- | --- | --- |
|  |  | **High (n, %)** | **Low (n, %)** |  |
| **Age (years)** |  | 53.88±10.82 | 53.13±12.17 | 0.8163 |
| **T stage** |  |  |  | 0.9874 |
| 1-2 | 38 | 20（52.6） | 18（47.4） |  |
| 3-4 | 8 | 4（50.0） | 4（50.0） |  |
| Tx | 4 | 2（50.0） | 2（50.0） |  |
| **N stage** |  |  |  | 0.3903 |
| 0-1 | 33 | 15（45.5） | 18（54.5） |  |
| 2-3 | 10 | 6（60.0） | 4（40.0） |  |
| Nx | 7 | 5（71.4） | 2（28.6） |  |
| **Ki67** |  |  |  | 0.7737 |
| <30% | 18 | 10（55.6） | 8（44.4） |  |
| ≥30% | 32 | 16（50.0） | 16（50.0） |  |
| **Hormone receptor** |  |  |  | 0.7851 |
| + | 26 | 13（50.0） | 13（50.0） |  |
| - | 24 | 13（54.2） | 11（45.8） |  |
| **Her2 positive** |  |  |  | 0.4674 |
| **+** | 9 | 6（66.7） | 3（33.3） |  |
| - | 41 | 20（48.8） | 21（51.2） |  |
| **Immune cell infiltration** |  |  |  | 0.7716 |
| yes | 19 | 9（47.4） | 10（52.6） |  |
| no | 31 | 17（54.8） | 14（45.2） |  |
| **MP grading system** |  |  |  | **0.0302** |
| 4-5 | 15 | 4（26.7） | 11（73.3） |  |
| 1-3 | 35 | 22（62.9） | 13（37.1） |  |

MP Grade, Miller–Payne (MP) grading system.
